# Long-term outcomes of balloon dilation of the eustachian tube under local anesthesia

**DOI:** 10.1007/s00405-026-10586-1

**Published:** 2026-09-17

**Authors:** Sean C. Sheppard, Iman Balvanovic, Sven Beckmann, Nathalie Fehre, Cecilia Lotto, Urs Borner, Lukas Anschuetz

**Affiliations:** 1 Department of Otorhinolaryngology, Head and Neck Surgery, Inselspital, Bern University Hospital, University of Bern, Bern, 3010 Switzerland; 2 Institut Pasteur, AP-HP, Inserm, CNRS, Fondation pour l’Audition, IHU reConnect, Technologies et thérapie génique pour la surdité, Université Paris Cité, Paris, F-75012 France; 3 Department of Otorhinolaryngology, Head and Neck Surgery, CHUV, University of Lausanne, Lausanne, 1011 Switzerland; 4 Department of Oral Surgery and Stomatology, School of Dental Medicine, University of Bern, Bern, 3010 Switzerland; 5 Department of Otolaryngology, Head and Neck Surgery, Technische Universität Dresden, Dresden, 01307 Germany; 6 The Sense Innovation and Research Center, Lausanne, 1007 Switzerland

**Keywords:** Eustachian tube dysfunction, Local anesthesia, In-office, Ear pressure, ETDQ-7, Tuboplasty

## Abstract

**Purpose:**

To evaluate the long-term outcomes, safety, and feasibility of balloon dilation of the Eustachian tube (BDET) performed under local anesthesia in patients with baro-challenge-induced and obstructive Eustachian tube dysfunction (ETD).

**Methods:**

This prospective observational cohort study included 65 adult patients (89 ears) undergoing in-office BDET under local anesthesia between May 2020 and June 2023. Symptoms were assessed using the 7-item Eustachian Tube Dysfunction Questionnaire (ETDQ-7) and a visual analogue scale (VAS). Clinical examination, Valsalva maneuver, tympanometry, and pure-tone audiometry were additionally evaluated when available. Follow-up was performed at 3, 12, and 24 months.

**Results:**

Thirty-seven patients had baro-challenge-induced ETD and 28 had obstructive ETD (OETD). All 65 patients underwent BDET under local anesthesia, with a mean intraoperative pain score of 5/10. Mean ETDQ-7 scores improved significantly in the overall cohort (p < 0.0001), with improvement maintained at 12 and 24 months. Improvement was significant in baro-challenge-induced ETD but not in OETD. Subjective ability to perform the Valsalva maneuver improved significantly in both groups, while air-conduction thresholds improved significantly in the baro-challenge group. One self-limiting cervicofacial subcutaneous emphysema occurred, with no major complications.

**Conclusion:**

In-office BDET under local anesthesia is a safe and feasible treatment for ETD, with symptomatic improvement maintained for up to 24 months. Patients with baro-challenge-induced ETD appear to benefit most, whereas outcomes in OETD are more variable. These findings highlight the importance of appropriate patient selection and counseling.

**Supplementary Information:**

The online version contains supplementary material available at https://doi.org/10.1007/s00405-026-10586-1.

## Introduction

Eustachian tube dysfunction (ETD) is a clinical condition characterized by impaired middle ear pressure homeostasis due to inadequate ventilation. The Eustachian tube plays a key role in pressure equalization, mucociliary clearance, and protection of the middle ear from pathogens and acoustic trauma. ETD is categorized into three main types: dilatory dysfunction, patulous dysfunction, and baro-challenge-induced dysfunction [1]. Dilatory dysfunction reflects an anatomical or functional obstruction, or a dynamic failure of the Eustachian tube to open adequately. Baro-challenged ETD occurs when the Eustachian tube fails to open properly in response to environmental pressure changes, such as during flying or diving, leading to symptoms like aural fullness, discomfort, and hearing difficulties [2]. Patulous dysfunction is characterized by an abnormally patent Eustachian tube, typically presenting with autophony. Diagnosis is based on clinical history and examination and may be supported by patient-reported outcome measures such as the Eustachian Tube Dysfunction Questionnaire (ETDQ-7) [3].

Management of ETD has traditionally involved medical treatments such as nasal decongestants, steroids, and auto inflation techniques, which aim to improve Eustachian tube patency and reduce symptoms. However, these treatments often yield inconsistent results [4]. In recent years, balloon dilation of the Eustachian tube (BDET) has emerged as a successful treatment option to conventional methods [5, 6]. Studies have shown a significant improvement for divers and aviators with baro-challenge-induced ETD in symptoms and a high return-to-duty rate after BDET [7, 8]. There are still remaining uncertainties surrounding the long-term effectiveness of BDET for patients with baro-challenged ETD, and further prospective studies are warranted [5]. This study aims to assess BDET under local anesthesia for patients with baro-challenged and obstructive ETD (OETD) with a long term follow up of 2 years.

## Materials and methods

This study was approved by the local ethics committee (KEK 2019-00555), as a prospective observational cohort study including patients undergoing in-office BDET between May 2020 and June 2023 at a otology/neurotology tertiary referral clinic. The study represents a continuation of our previously published work [9].

Indication for BDET was placed for patients who suffered from ETD and were refractory to medical management including topical nasal steroid therapy and auto inflation for at least 3 months. Potential risk factors including allergy, reflux and sleep apnea were treated before proposing the surgical intervention. Diagnosis was based on the presence of symptoms such as pressure imbalances, aural fullness, popping, and discomfort/pain, in combination with nasopharyngeal endoscopy findings suggestive of intraluminal edema of the eustachian tube. Baro-challenged patients were defined as patients suffering from symptoms during changements of atmospheric pressure. Other patients were classified as OETD. Patients with autophony and suspected of patulous ETD were excluded, as BDET is not indicated for this condition. Patients younger than 18 years were excluded.

Preoperative assessment included medical history, evaluation of risk factors, and clinical examination. Subjective and objective Valsalva maneuvers were evaluated. Tympanometry and pure tone audiometry were performed. Computer tomography was not routinely performed. Symptom severity was assessed using ETDQ-7 questionnaire and a visual analog scale (VAS) of symptom discomfort.

The surgical technique under local anesthesia has been previously described [9]. The type of local anesthesia, adherence to local anesthesia, duration of anesthesia preparation, and surgical time were recorded. Directly after the intervention, patients were asked to rate intraoperative pain using a visual analogue scale (VAS) and to indicate whether they would recommend undergoing the procedure under local anesthesia rather than general anesthesia.

Follow-up assessments were conducted at 3, 12, and 24 months. At 3 months, patients underwent clinical examination, ETDQ-7 and VAS assessment, and audiometric testing. Some patients living far from the hospital were seen by external ORL specialists for their follow-up at 3 months and were contacted by telephone for ETDQ-7 and VAS scores. Patient satisfaction was also evaluated using a VAS. For patients with baro-challenged ETD, additional questions assessed symptom characteristics, including duration and frequency. At 12 and 24 months, follow-up was conducted by telephone, with reassessment of ETDQ-7 and VAS scores.

### Statistical analysis

Statistical analysis was performed and plotted using GraphPad Prism 11 (GraphPad Software, Inc., San Diego, CA, United States). Unless otherwise specified, data were analyzed at the patient level. Ear-level analyses were limited to otoscopic findings, objective Valsalva maneuver, and audiometry and tympanometry data. Comparisons of paired, normally distributed continuous variables were performed using the paired t-test, while non-normally distributed or ordinal data were analyzed using the Wilcoxon matched-pairs signed-rank test. Paired categorical variables were analyzed using the McNemar test. Unpaired continuous variables were compared using the unpaired t-test and Fisher’s exact test was used for categorical variables. Audiometric analyses were conducted when both pre- and postoperative data were available. All tests were two-sided, and statistical significance was defined as *P* < 0.05.

## Results

### Demographic and clinical characteristics

The study cohort consisted of 65 patients (89 ears) with a mean age of 44 years (range 18–79), including 24 women and 41 men, who underwent surgery in local anesthesia for ETD (Table 1). Thirty-seven patients presented with baro-challenged ETD and 28 with OETD. No differences in preoperative clinical or demographic characteristics were observed between the groups.

**Table 1:** Patient clinical and demographic characteristics

	Baro-challenged ETD (*n* = 37)	Obstructive ETD (*n* = 28)
Age	39,5 [20–79]	41,4 [18–78]
Gender		
Female	17 (46%)	7 (25%)
Male	20 (54%)	21 (75%)
Affected side		
Right / Left	11 (30%) / 12 (32%)	9 (32%) / 9 (32%)
Bilateral	14 (38%)	10 (36%)
Onset of symptoms		
Sudden	14 (38%)	14 (50%)
Progredient	23 (62%)	14 (50%)
Audiometric Data		
Mean air conduction (dB)	18 ± 15.0 (42)	19 ± 14.6 (31)
Mean bone conduction (dB)	11 ± 12.3 (41)	10 ± 10.2 (30)
Mean Airbone Gap (dB)	7 ± 7.7 (41)	9 ± 8.6 (30)
Tympanogram	*n* = 35	*n* = 30
A	23 (66%)	14 (47%)
B	5 (14%)	12 (40%)
C	7 (20%)	4 (13%)
Risk Factors		
Smoking		
Active smoking	7 (19%)	7 (25%)
No smoking / past	30 (81%)	21 (75%)
Reflux	4 (11%)	2 (7%)
Allergies	7 (19%)	6 (21%)
Sleep apnea syndrome	3 (8%)	4 (14%)
BMI	24.9 ± 4.79 [17.4–37.0] (32)	25.1 ± 3.46 [19.1–34.3] (19)
Previous adenoid surgery	20 (54%)	17 (61%)

Comparison between baro-challenged and chronic obstructive ETD (Eustachian tube dysfunction) groups showed no differences. Categorical variables were analyzed using Fisher’s exact test, and continuous variables using the unpaired t-test. Data of audiometric data and tympanograms were calculated per ear

Data are presented as median, mean ± standard deviation with range [min–max]. The number of observations is indicated (n) where data were missing

### Intraoperative data

Local anesthesia adherence was achieved in 65 patients. For one patient, initially planned for both sides, the surgery was completed unilaterally, while the contralateral side was aborted due to pain related to septal deviation. Another patient aborted the intervention before nasal endoscopy due to a high level of anxiety and narrow nasal anatomy and was excluded from the analysis. In both cases, the procedure was subsequently performed under general anesthesia. Local anesthesia was administered as topical spray in 53 patients, infiltration alone in one patient, and a combination of spray and infiltration in 11 patients. The mean local anesthesia preparation time was 16 ± 1.9 min (range 15–25). Mean surgical time was of 6 ± 4.3 min (range 3–25). Mean intraoperative pain using a visual analogue scale (0 = no pain, 10 = maximum pain) was 5 ± 2.2. All sixty-five patients who completed the procedure in local anesthesia reported that they would recommend performing the procedure in the in-office setting and were satisfied with the intervention. The patient with partial adherence of the local anesthesia also recommended to undergo the surgery in local anesthesia for the successful side.

### ETD symptoms pre- and postoperative

Overall, the mean ETDQ-7 score decreased from 3.0 ± 1.10 preoperatively to 2.4 ± 1.09 at 3 months postoperatively (*p* < 0.0001). This improvement was maintained at 12 months (2.5 ± 1.09) and 24 months (2.24 ± 0.89). Subgroup analysis (Table 2) showed a significant reduction in ETDQ-7 scores in the baro-challenged ETD group, with a decrease from 3.0 to 2.2 (*p* < 0.0001), whereas the reduction observed in the OETD group was not significant (*p* < 0.06). ETDQ-7 scores remained stable between 3 and 12 months and between 12 and 24 months in both groups.

**Table 2:** Preoperative and postoperative mean ETDQ-7 and VAS scores in baro-challenged Eustachian Tube Dysfunction (ETD) and obstructive Eustachian tube dysfunction (OETD)

	Preoperative		Postoperative 3 mo		Postoperative 12 mo		Postoperative 24 mo	
	Baro ETD (37)	OETD (28)	Baro ETD (37)	OETD (28)	Baro ETD (29)	OETD (16)	Baro ETD (18)	OETD (9)
ETDQ7 score	3.0 ± 1.08 [1.3–4.9]	3.0 ± 1.14 [1.3–5.4]	2.2 ± 0.97 [1-6.6] **	2.6 ± 1.19 [1-5.1]	2.3 ± 1.04 [1-5.1]	2.8 ± 1.11 [1–5]	2.1 ± 1.01 [1-4.7]	2.6 ± 0.49 [1.9–3.6]
VAS score	5.8 ± 2.81	5.6 ± 2.76	4.7* ± 2.80	4.5* ± 2.65	4 ± 2.83	4.0 ± 2.92	3.7 ± 2.83	3.7 ± 2.83

Comparison between preoperative and 3-month ETDQ-7 and mean VAS symptom scores (0 = no discomfort, 10 = maximum discomfort) in both baro-challenged and obstructive Eustachian tube dysfunction groups was performed with paired t-test. No differences were observed in mean ETDQ-7 or VAS symptom scores between 3 and 12 months or between 12 and 24 months

*p < 0.05; **p < 0.0001

*Abbreviations*: *ETDQ-7* Eustachian Tube Dysfunction Questionnaire-7, *VAS* Visual analog score

Analysis of individual ETDQ-7 items (Supplementary Table 1) showed that symptoms of pressure, pain, feeling of clogged ears, crackling or popping sounds, and muffled hearing were most improved postoperatively in the baro-challenged group. In contrast, in the OETD group, improvement was observed exclusively for feeling of clogged ears. Baro-challenged patients most frequently reported symptom occurrence during leisure activities (92%), followed by symptoms occurring at home (22%) and at work (19%). Regarding symptom characteristics in baro-challenged ETD (Supplementary Table 2), a reduction in symptoms was observed overall, particularly in situations involving pressure changes such as air travel and mountain activities related to high-altitude. Symptom duration decreased postoperatively, whereas symptom frequency did not change significantly.

Subjective ability to perform the Valsalva maneuver increased in both groups. In the baro-challenged ETD group, the proportion of patients able to perform the maneuver increased from 2 (5%) to 24 (65%) (McNemar test, *p* < 0.0001). In the OETD group, this increased from 4 (14%) to 15 (54%) (McNemar test, *p* < 0.01). Objective Valsalva by ear assessed by otoscopy improved overall from 40 (64%) preoperatively to 51 (81%) postoperatively (data available for 63 ears; McNemar, *p* < 0.04), with no significant differences in the subgroup analysis. At 3 months, patient-reported satisfaction (VAS) was 7.2 ± 1.93 in the baro-challenged group and 6.7 ± 2.14 in the OETD group, with no significant difference between groups.

A subgroup analysis restricted to patients who underwent bilateral balloon dilation demonstrated significant improvements in both symptom severity and quality of life at 3 months. Mean ETDQ-7 scores decreased from 2.9 ± 1.21 to 2.3 ± 0.95 (paired t-test, *p* < 0.05) and mean VAS decreased from 5.4 ± 2.6 to 3.6 ± 2.64 (paired t-test, *p* < 0.05).

### Audiometric data

Pure-tone audiometry with air conduction threshold was available for 73 of 89 ears, bone conduction and air–bone gap data was available for 71 ears. In the baro-challenged ETD group, a significant improvement in air conduction thresholds was observed postoperatively (18 ± 14.9 dB preoperatively vs. 16 ± 17.3 dB postoperatively; Wilcoxon test, *p* < 0.05). No significant changes were found in bone conduction thresholds or air–bone gap. In the OETD group, no significant changes were observed in air conduction thresholds, bone conduction thresholds, or air–bone gap between preoperative and postoperative measurements.

Tympanometry data were available for 60 ears. Patients presenting a tympanic membrane perforation were excluded from this analysis. In a dichotomized analysis (type A vs. type B/C), no significant difference was observed between the preoperative and postoperative findings (McNemar test, *p* < 0.5).

### Adverse effects

One case of self-limiting postoperative cervicofacial subcutaneous emphysema was diagnosed clinically and radiologically, which was managed conservatively with prophylactic antibiotics and resolved without further complications. No major complications occurred.

## Discussion

In this study, we evaluated the outcomes of BDET performed under local anesthesia in patients with ETD, comparing patients with baro-challenged ETD and OETD. Our findings show a significant improvement in mean ETDQ-7 score in the baro-challenged ETD group with effects observed at 3 months remained stable throughout the 24-month follow-up period, which is the study with the longest follow-up reported.

Performing BDET under local anesthesia proved to be safe and efficient, in line with the literature [10, 11]. Most described local anesthesia protocols combine topical decongestants with liquid local anesthetics, followed by additional gel or creme of local anesthetics. Møller reported his experience with 1001 BDET procedures in 511 patients using three different protocols [12]. While his initial protocol was similar to ours, with a reported mean intraoperative pain score of of 6.7 on the VAS, his third protocol consisted of premedication with benzodiazepine and a sphenopalatine ganglion block which reduced the pain score to 1.4 on the VAS. In our cohort, moderate intraoperative pain was reported, and patients were satisfied with the procedure and would recommend the procedure in local anesthesia. Nevertheless, some patients with higher pain sensitivity may benefit of a more invasive anesthesia protocol to further improve comfort. Particular attention should be paid to patient selection, especially regarding nasal anatomy, as anatomical constraints limited the procedure in two of our cases. In addition, performing BDET in an office-based setting may reduce healthcare costs by avoiding general anesthesia, operating room time, additional surgical staff, and postoperative monitoring.

Baro-challenged ETD is characterized by symptoms occurring during changes in ambient pressure. These patients may present subclinical forms of ETD with normal objective test results under stable pressure conditions [13, 14]. In our study, objective assessments such as pressure chambers or tubomanometry were not available, as it is the case in most centers internationally. The ETDQ-7 questionnaire, although subjective, is a validated tool and while not specific for baro-challenged ETD, has been used in studies with baro-challenged ETD. Our results show a significant improvement of the ETDQ-7 score which last up to the follow-up of 24 months, correlating with the literature [3, 5, 15]. Although patients in our cohort had lower preoperative ETDQ-7 scores than those included in the systematic review by Jansen et al. (21 ± 7 vs. 31 ± 6), postoperative scores were comparable between studies (16.8 ± 8 in our cohort vs. 12 ± 6 in the review) [15]. Thus, despite a smaller absolute range of improvement, the final symptom burden achieved after treatment was similar. Moreover, the clinical relevance of this improvement is supported by the concomitant reduction in VAS symptom scores and the high levels of patient satisfaction observed throughout follow-up. While most studies report specific populations of aviators or divers, our cohort mainly consisted of patients experiencing symptoms during leisure activities involving altitude changes, such as travelling by plane or activities in mountainous regions, which is common in our country. In our cohort, 41% of patients reported complete resolution of symptoms at 3 months. The remaining patients experienced a reduction in symptom duration. Our results support the effectiveness of BDET in baro-challenged ETD.

While ETDQ-7 scores improved in the overall cohort, subgroup analysis revealed a reduction but with no statistical significance in patients with OETD following BDET. This finding may be partly explained by the presence of patients with adhesive otitis media, who are known to respond less favorably to this intervention [16]. Sandoval et al. [17], categorized their ETD cohort into baro-challenged, chronic serous otitis media and adhesive otitis media. They reported that baro-challenged ETD benefited the most from BDET, followed by chronic serous otitis media. In contrast, patients with adhesive otitis media had an improvement of ETDQ-7 and tympanogram not reaching statistical significance, consistent with our results. A large proportion of studies report their results in a group of “chronic ETD” populations without distinguishing baro-challenged ETD, which may introduce bias and overestimate the overall treatment effect [18]. Our findings support that patients presenting baro-challenged ETD benefit most from BDET, whereas outcomes in other forms of ETD remain variable. In consequence, patients without obvious baro-challenged symptoms or identifiable etiologies should be counseled against a surgical intervention or at least regarding an uncertain and potentially limited benefit of the procedure.

While the prospective design of the study represents a strength, several limitations of our study should be considered. The study was designed as an observation study without a control group. Unfortunately, not all patients underwent follow-up tympanometry or audiometry, as some follow-up assessments were performed externally and were not available for analysis. At the 24-month follow-up, we report a substantial attrition which may limit long term assessment. Furthermore, part of the follow-up was conducted by telephone, which may introduce reporting bias. In addition, symptom assessment relied primarily on questionnaire-based measures. Although the ETDQ-7 is a validated questionnaire, it remains subjective to recall bias.

## Conclusion

BDET performed under local anesthesia is a safe, feasible, and cost-effective procedure. It appears to be particularly effective in patients with baro-challenged ETD, whereas outcomes in patients with OETD are more variable. These findings underscore the importance of careful patient selection and appropriate patient counseling.

## Supplementary Information

Supplementary Material 1.

## Data Availability

Anonymized version of the data is available upon request.
