# Supplementary material for: Long-term outcomes of balloon dilation of the eustachian tube under local anesthesia
Source: Eur Arch Otorhinolaryngol. 2026 Sep 17;283(12):7869–74. doi: 10.1007/s00405-026-10586-1 (PMC13768368; doi:10.1007/s00405-026-10586-1)
Supplement: Supplementary file 1 [file 405_2026_10586_MOESM1_ESM.docx]

**Supplementary Table 1. Individual ETDQ-7 item scores preoperatively and at 3 months postoperatively in baro-challenged and obstructive Eustachian tube dysfunction patients (OETD)**

|  | Preoperative | | Postoperative 3 mo | | |
| --- | --- | --- | --- | --- | --- |
|  | Baro (37) | OETD (28) | Baro (37) | OETD (28) |  |
| Pressure | 3.8 ± 1.42 | 3.6 ± 1.69 | 2.6 ± 1.24**** | 3.3 ± 1.82 |  |
| Pain | 2.4 ± 1.74 | 2.2 ± 1.19 | 1.6 ± 0.95** | 1.9 ± 1.46 |  |
| Clogged ears | 3.6 ± 1.59 | 4.4 ± 1.99 | 2.6 ± 1.48*** | 3.4 ± 1.53** |  |
| Worsening during cold or sinusitis | 2.3 ± 1.72 | 2.4 ± 1.83 | 1.9 ± 1.69 | 2.1 ± 1.61 |  |
| Crackling or popping sounds | 3.0 ± 1.69 | 3.0 ± 2.14 | 2.2 ± 1.41** | 2.6 ± 1.93 |  |
| Ringing | 2.3 ± 1.74 | 2.79 ± 1.99 | 1.9 ± 1.49 | 2.2 ± 1.77 |  |
| Muffled Hearing | 3.4 ± 1.74 | 3.3 ± 1.67 | 2.3 ± 1.35*** | 3.0 ± 1.95 |  |

Comparison between preoperative and 3-month single items of the ETDQ-7 score was performed with paired t-test.
*p<0.05, ** p<0.01, *** p<0.001, ****p<0.0001

**Supplementary Table 2. Symptom characteristics in patients with baro-challenged Eustachian tube dysfunction before and at 3 months postoperatively**

|  | Preoperative n (%) | Postoperative 3 mo n (%) |
| --- | --- | --- |
| Symptoms during pressure change ^a^ | 37 (100%) | 22 (59%)*** |
| Triggering Situation ^a^ |  |  |
| Plane | 23 (62%) | 12 (32%)** |
| Mountain | 30 (81%) | 18 (49%)** |
| Diving | 5 (14%) | 4 (11%) |
| Tunnel | 1 (3%) | 3 (8%) |
| Maximum symptom duration ^b^* |  |  |
| Seconds | 6 (16%) | 4 (11%) |
| Minutes | 12 (33%) | 9 (24%) |
| Hours | 3 (8%) | 3 (8%) |
| Days | 13 (35%) | 6 (16%) |
| Months | 3 (8%) | 0 |
| Maximum symptom frequency ^b^ |  |  |
| Daily | 7 (19%) | 4 (11%) |
| Weekly | 8 (22%) | 5 (14%) |
| Monthly | 13 (35%) | 10 (27%) |
| Yearly | 9 (24%) | 3 (8%) |

^a^ Comparisons was performed using McNemar test. For triggering situations, multiple responses were allowed.

^b^ Comparisons of paired preoperative and postoperative data were performed using the Wilcoxon matched-pairs signed-rank test.

*p<0.05; ** p<0.01; ***p<0.001
